# The cellular response to vascular endothelial growth factors requires co-ordinated signal transduction, trafficking and proteolysis

**DOI:** 10.1042/BSR20150171

**Published:** 2015-09-29

**Authors:** Gina A. Smith, Gareth W. Fearnley, Darren C. Tomlinson, Michael A. Harrison, Sreenivasan Ponnambalam

**Affiliations:** * School of Molecular & Cellular Biology, University of Leeds, Leeds LS2 9JT, U.K.; † School of Biomedical Sciences, University of Leeds, Leeds LS2 9JT, U.K.

**Keywords:** disease, drugs, isoforms, membrane trafficking, receptor tyrosine kinase, signal transduction, vascular endothelial growth factor (VEGF)

## Abstract

VEGFs (vascular endothelial growth factors) are a family of conserved disulfide-linked soluble secretory glycoproteins found in higher eukaryotes. VEGFs mediate a wide range of responses in different tissues including metabolic homoeostasis, cell proliferation, migration and tubulogenesis. Such responses are initiated by VEGF binding to soluble and membrane-bound VEGFRs (VEGF receptor tyrosine kinases) and co-receptors. VEGF and receptor splice isoform diversity further enhances complexity of membrane protein assembly and function in signal transduction pathways that control multiple cellular responses. Different signal transduction pathways are simultaneously activated by VEGFR–VEGF complexes with membrane trafficking along the endosome–lysosome network further modulating signal output from multiple enzymatic events associated with such pathways. Balancing VEGFR–VEGF signal transduction with trafficking and proteolysis is essential in controlling the intensity and duration of different intracellular signalling events. Dysfunction in VEGF-regulated signal transduction is important in chronic disease states including cancer, atherosclerosis and blindness. This family of growth factors and receptors is an important model system for understanding human disease pathology and developing new therapeutics for treating such ailments.

## INTRODUCTION

The VEGF (vascular endothelial growth factor) superfamily and its receptors are highly conserved in metazoan species [1]. In lower eukaryotes, such as worms and flies, a hybrid PDGF (platelet-derived growth factor) and VEGF system of ligands and receptors mediates a range of biological responses. Many of the VEGF genes and receptors play essential roles in animal development and function. However, a recurring theme is the subversion of VEGF ligand and receptor function in a range of pathologies including cancer, atherosclerosis, AMD (age-related macular degeneration) and pre-eclampsia. In the present review, we will consider how ligand and receptor diversity underpins complexity in signal transduction, membrane trafficking and proteolysis with implications for normal and pathophysiological states.

## VEGF DIVERSITY AND FUNCTIONALITY

The VEGF superfamily consists of five structurally-related members of angiogenic and lymphangiogenic polypeptides: VEGF-A, VEGF-B, VEGF-C, VEGF-D and PlGF (placental growth factor). These growth factors are highly conserved with subtle differences in the distribution of charged residues determining receptor binding specificity (Figure 1). VEGFs regulate blood and lymph vessel development in an isoform-specific manner through activation of class III receptor tyrosine kinases (VEGFRs): VEGFR1 [Flt-1 (fms-related tyrosine kinase 1)], VEGFR2 [KDR (kinase insert domain receptor)] and VEGFR3 (Flt-4) [2]. VEGF polypeptides form homodimers but heterodimers of VEGF-A and PlGF also occur [3]. Complexity in the VEGF family is heightened by alternative splicing of VEGF-A, VEGF-B and PlGF and proteolytic processing of VEGF-C and VEGF-D. This allows multiple protein isoforms with distinct receptor and extracellular matrix-binding properties to be encoded by a single gene [4]. Correct *VEGFA* gene dosage is essential for normal mammalian health and development. Heterozygous *VEGFA^+/−^* knockout mice die between embryonic days E11 and E12 due to a deformed vascular network [5,6]. Dysfunction in the response to VEGF-A can cause pathological angiogenesis and play pivotal roles in chronic inflammatory diseases, ischaemic heart disease, cancer and retinopathy [7–9].

**Figure 1:**
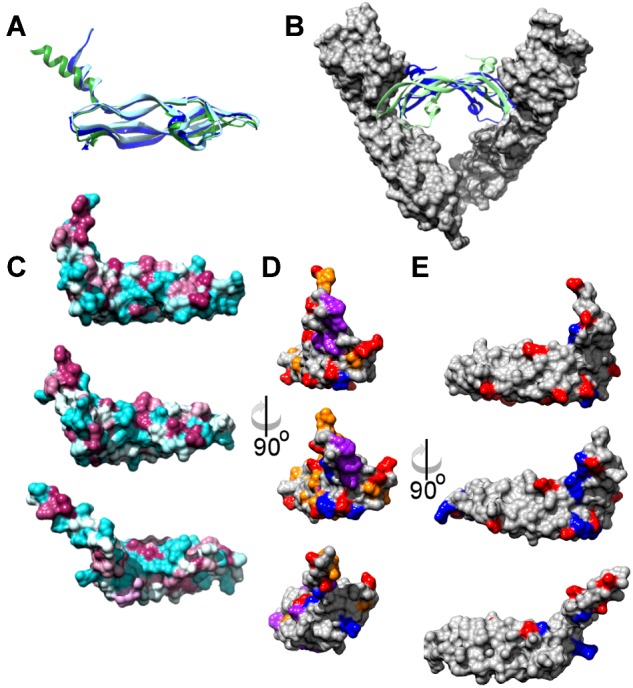
Structural differences between VEGF-A, PlGF and VEGF-C determine VEGFR binding (**A**) Ribbon diagram depicting the structural similarities between VEGF-A (blue; PDB ID: 3V2A), PlGF (green; PDB ID: 1RV6) and VEGF-C (cyan; PDB ID 4BSK). (**B**) A model of VEGF-A binding to VEGFR2 using the PlGF dimer as a template for VEGF-A binding to the Ig-like domains. (**C**) Structures of VEGF-A (top), PlGF (middle) and VEGF-C (bottom) reveal that although the fundamental fold is similar, the distribution of hydrophobic (purple) and polar (cyan) residues highlights differences between VEGFR1-binding ligands, VEGF-A and PlGF and VEGFR3-binding ligand, VEGF-C. (**D**) Structures of VEGF-A (top), PlGF (middle) and VEGF-C (bottom) rotated 90° with positive (blue), negative (red), aliphatic (yellow) and aromatic (purple) residues highlighted. (**E**) Structures of VEGF-A (top), PlGF (middle) and VEGF-C (bottom) rotated 90° with positive (blue) and negative (red) residues highlighted.

The human *VEGFA* gene encodes a pre-mRNA with at least eight exons and seven introns [10]. Alternative RNA splicing produces at least seven pro-angiogenic isoforms of human VEGF-A which encode polypeptides of 121, 145, 148, 165, 183, 189 or 206 residues (a isoforms) and five anti-angiogenic isoforms of 121, 145, 165, 183 and 189 residues denoted VEGF-A_xxxb_. Recent work has shown that *VEGFA* mRNA also undergoes programmed translational read-through to generate an anti-angiogenic VEGF-A_x_ isoform containing a unique 22 amino acid C-terminus extension [11]. Each VEGF-A isoform contains exons 1–5 which encode the signal sequence (exon 1), N-terminus dimerization domain (exon 2), VEGFR1-binding and N-glycosylation site (exon 3), VEGFR2-binding site (exon 4) and a plasmin cleavage site (exon 5). Exons 6a, 6b, 7a and 7b encode the heparin-binding domain and their variable inclusion significantly influences the biological properties of each VEGF-A isoform. Those isoforms containing exon 6a, such as VEGF-A_145_ and VEGF-A_189_, are weaker chemotactic cytokines and mitogens [12–14]. Exon 6a has a preponderance of basic amino acids which act to directly reduce VEGFR2–VEGF-A binding [15]. Interestingly, exon 6-containing isoforms do not inhibit VEGF-A-stimulated VEGFR1 activity and can promote VEGFR1-mediated vascular permeability [14,16].

Signal transduction and protein kinase activity is implicated in regulating proximal and distal splice site selection on the primary RNA, e.g. specifying the C-terminus six amino acids with either the pro-angiogenic CDKPRR (exon 8a) or anti-angiogenic SLTRKD (exon 8b) sequence [17]. The C-terminus SLTRKD sequence in the anti-angiogenic VEGF-A_165b_ isoform cannot bind the co-receptor, NRP1 (neuropilin 1), leading to an altered VEGFR2 protein complex which exhibits reduced tyrosine kinase activity [17]. Reduced co-receptor binding could explain the anti-angiogenic properties of VEGF-A_165b_ in combination with competition between VEGF-A_165b_ and pro-angiogenic VEGF-A_165a_ isoforms for binding to VEGFR2 [13,17]. Down-regulated VEGF-A_165b_ expression correlates with cellular switching to a pro-angiogenic phenotype that is associated with multiple pathologies including diabetic retinopathy and several adult epithelial cancers [18,19]. Conversely, up-regulated VEGF-A_165b_ expression in skin and circulatory tissues of patients with systemic sclerosis hinders angiogenesis and vascular repair [20].

Human *VEGFB* contains seven exons and encodes at least two isoforms with alternative splice acceptor sites present in exon 6 [21,22]. The VEGF-B_167_ C-terminus contains the highly basic NRP1/heparin-binding domain whereas the more freely diffusible VEGF-B_186_ isoform has a hydrophobic C-terminus which undergoes O-linked glycosylation and proteolytic processing [21].

Within the VEGF family, VEGF-C and VEGF-D are unique in being initially synthesized as precursor proteins containing long N- and C-terminal propeptides [23,24]. Proteolytic removal of both the N- and the C-propeptides releases mature, bioactive VEGF-D containing the central VEGF-homology domain. Such processing increases VEGF-D affinity for VEGFR3; furthermore, only mature VEGF-D binds VEGFR2 [25]. Although two mouse VEGF-D isoforms have been described [26], little is known about alternate RNA splicing of human VEGF-C and VEGF-D.

Four PlGF isoforms with distinct properties are encoded by the *PGF* gene. The most commonly expressed or major isoforms are PlGF-1 (131 amino acids) and PlGF-2 (152 amino acids) [27]. The PlGF-2 exon 6 heparin-binding domain facilitates binding to heparin and NRP1. Contrastingly, PlGF-1 and PlGF-3 (203 amino acids) lack exon 6 so are unable to bind heparin [28]. PlGF-3 contains an additional 216 nt insertion between exons 4 and 5. PlGF-4 (224 amino acids) consists of the same sequence as PlGF-3 plus the exon 6-encoded heparin-binding domain [29]. These larger isoforms may function similarly to VEGF-A_189_ and VEGF-A_206_ [29].

Notably, crystal structures of VEGFs lack information on the C-terminal portion of these proteins (Figure 1). This raises important functional questions as to how binding by the VEGF carboxy proximal domain(s) ‘programs’ assembly of a functional signalling complex with different molecular partners recruited depending on the VEGF isoform and VEGFR involved.

VEGFs bind to the extracellular domain of VEGFRs and additional cell surface-expressed co-receptors, e.g. HSPGs (heparan sulfate glycoproteins), NRPs, integrins and ephrin B2 [30,31]. PlGF and VEGF-B specifically bind VEGFR1 and NRP1 whereas VEGF-A binds both VEGFR1 and VEGFR2 (Figure 2) [32–34]. VEGF-C and VEGF-D bind VEGFR2 and VEGFR3 (Figure 2) [35]. Distinct splice variants of VEGF-A assemble specific receptor–co-receptor complexes. Spatial and temporal aspects of VEGFR signal transduction can be influenced by the restricted diffusion of HS-binding VEGFs and is further modulated by VEGF interactions with the extracellular matrix [36]. Binding of VEGF-A_165a_ and VEGF-A_189_ to HSPGs and NRP1 promotes ternary complex formation and VEGFR2 signal transduction. NRP1 binding to VEGF-A_165a_ enhances VEGFR2–VEGF-A_165a_ complex formation and tyrosine kinase activity. Optimal p38 MAPK (mitogen-activated protein kinase) activation is achieved through NRP1-enhanced VEGF-A_165a_ signal transduction [13]. In contrast, VEGF-A_121_ is freely diffusible but NRP1 binding does not promote ternary complex formation with VEGFR2, causing reduced signal transduction [37]. Despite PlGF only binding VEGFR1, activation of VEGFR2 could occur indirectly through VEGF-A displacement from VEGFR1, thus increasing VEGF-A bioavailability for VEGFR2 [38]. Furthermore, PlGF/VEGF-A heterodimers can induce VEGFR1/VEGFR2 dimerization and downstream VEGFR2 activation [39].

**Figure 2:**
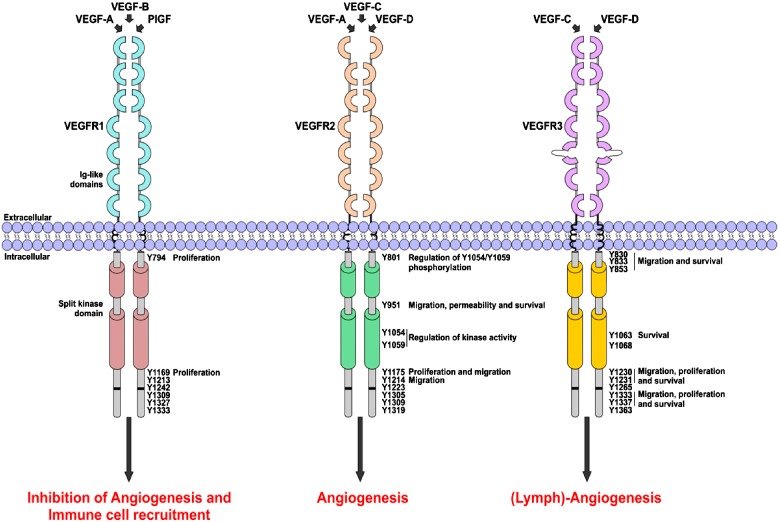
Receptor tyrosine kinase subfamily of VEGFR proteins Schematic illustrating interacting VEGF ligands, cytoplasmic phosphorylated tyrosine residues and functional outputs associated with each VEGFR upon VEGF binding, Ig-like domains mediate interaction between VEGFR monomers to promote complex assembly.

The substantially different functions of VEGFR1-specific ligands, VEGF-B and PlGF, raise the possibility that VEGFR1 function is regulated by co-receptor recruitment and/or cell-specific intracellular signalling events [40]. VEGF-B-stimulated fatty acid synthesis in endothelial cells is crucial in organs which experience high metabolic stress, such as the heart, and involves both VEGFR1 and NRP1 activation [41]. Such therapeutic aspects are highlighted in cardiac endothelial cells where VEGF-B promotes physiological angiogenesis and revascularization of the ischaemic myocardium [42]. In contrast, PlGF expression is associated with cancer progression and required for inflammation-associated angiogenesis [38]. PlGF promotes pathological angiogenesis in several inflammatory disease states in which VEGFR1-regulated recruitment of bone marrow-derived monocytes precedes deposition of angiogenic growth factors [38]. Tumours that exhibit increased PlGF secretion suggest a functional link between VEGFR1 activity and cancer progression.

## VEGFR DIVERSITY AND FUNCTIONALITY

The RTK (receptor tyrosine kinase) subfamily containing VEGFRs is denoted as class V. These membrane-bound RTKs comprise VEGFR1, VEGFR2 and VEGFR3. VEGFRs exhibit structural and sequence homologies and comprise an extracellular ligand-binding domain consisting of seven Ig-like repeats, a transmembrane domain, a juxtamembrane domain, a split tyrosine kinase domain and a C-terminal tail.

### VEGFR1

The *VEGFR1* gene contains 30 exons and encodes an estimated 151 kDa transmembrane receptor which undergoes post-translational modifications to produce a ∼180 kDa mature glycoprotein [43,44]. VEGF-A has highest affinity for VEGFR1 [44] but the activated complex exhibits relatively weak tyrosine kinase activity and forms a non-productive signalling complex [10,45]. This poor tyrosine kinase activity of VEGFR1 is thought to arise from structural properties of the activation loop, a repressor sequence in its juxtamembrane domain and a lack of positive regulatory tyrosine residues [46–48]. VEGFR1 is expressed in various cell types including both quiescent and actively proliferating endothelial cells, haematopoietic stem cells, monocytes, macrophages and tumour cells [2,10,40]. VEGFR1 is essential for mammalian development as homozygous *VEGFR1*^−/−^ knockout mice die between embryonic days E8.5 and E9.5 after endothelial hyperproliferation leads to blood vessel obstruction [49]. The *VEGFR1* primary RNA transcript also undergoes alternative splicing to generate a soluble VEGFR1 isoform (sFlt-1; sVEGFR1) of ∼110 kDa. This sVEGFR1 isoform comprises Ig-like domains 1–6 of the VEGFR1 ectodomain but also includes a unique 31 residue sequence (encoded by intron 13) at the C-terminus [50]. This sVEGFR1 can be a potent inhibitor of VEGF-A, VEGF-B and PlGF signal transduction [51]. In leukaemia cells, PlGF and VEGF-A induce tyrosine phosphorylation of VEGFR1 and increase ectodomain shedding. This occurs via PKC (protein kinase C) activation and metalloproteases such as TACE [tumour necrosis factor α (TNFα) converting enzyme] [52]. TACE activity generates sVEGFR1 and an intracellular cytoplasmic fragment; plasma membrane detachment of this cytoplasmic fragment requires γ-secretase/presenilin activity [53].

One view is that VEGFR1 has positive or negative regulatory roles in angiogenesis depending on biological conditions. A VEGFR1 negative regulatory role is thought to arise from sVEGFR1 acting as a decoy receptor to sequester VEGF-A away from VEGFR2 or by formation of non-signalling VEGFR1–VEGFR2 heterodimers [54]. A VEGFR1 positive regulatory role could occur under pathological conditions of tumour growth; here, abnormally high expression of VEGFR1-specific ligands leads to elevated VEGFR1 tyrosine kinase activity and promotes angiogenesis [55].

### VEGFR2

Immature VEGFR2 has an estimated molecular mass of ∼152 kDa and undergoes translocation to the endoplasmic reticulum and N-linked glycosylation along the secretory pathway to produce a mature glycoprotein with an approximate mass of 200–230 kDa [36,45]. Only mature fully glycosylated VEGFR2 undergoes efficient *trans*-autophosphorylation following VEGF stimulation [56]. Alternative splicing generates sVEGFR2, which is present in plasma, as well as in multiple tissues, including the heart, spleen, skin, ovary and kidney. This sVEGFR2 can sequester free VEGF-C, thus preventing VEGFR3 activation and inhibiting lymphatic endothelial cell proliferation [57]. VEGFR2 is essential for healthy mammalian development as homozygous *VEGFR2*^−/−^ knockout mice die at embryonic day E8.5, exhibiting impaired haematopoietic and endothelial cell development leading to formation of an insufficient vascular network [58]. This also mimics the *VEGFA^+/−^* knockout mice phenotype [5,40,59]. VEGFR2 expression is predominantly restricted to endothelial cells and haematopoietic stem cell precursors, with peaks in expression during embryonic development [60].

VEGF-A binds VEGFR2 with a relatively high affinity (*K*_d_ ∼ 150 pM); however, this parameter is ∼10-fold lower than that for VEGFR1 (*K*_d_ ∼ 15 pM) [61]. Nonetheless, the majority of VEGF-A-regulated angiogenesis effects are attributed to interaction with VEGFR2. One view is that VEGFR2 is a more potent tyrosine kinase which targets numerous substrates including membrane proteins, cytoplasmic enzymes and regulators [2,62]. VEGFR2 is thus considered to be the major pro-angiogenic switch which regulates blood vessel development and homoeostasis in response to circulating VEGFs [2,62]. VEGFR2 expression is down-regulated in quiescent adult vascular network [63], probably reducing the magnitude of VEGFR2-regulated pro-angiogenic responses [64].

### VEGFR3

VEGFR3 is an essential regulator of lymphoendothelial function and lymphangiogenesis. Upon co-translational insertion of newly synthesized VEGFR3 into the endoplasmic reticulum, this ∼195 kDa precursor protein undergoes N-linked glycosylation and proteolytic cleavage within the fifth Ig-like domain. This generates an N-terminal polypeptide which forms a stable disulfide linkage with the carboxy half of the VEGFR3 precursor [65]. VEGFR3 complexity is further increased by alternative splicing to produce both long and short isoforms [66,67]. The VEGFR3 short isoform lacks 65 residues proximal to the C-terminus; this is only present in humans and probably arose through a retroviral integration event during human speciation [68]. Furthermore, the VEGFR3 short isoform lacks two carboxy proximal cytoplasmic phosphorylation epitopes which are detected in VEGFR3 homodimers but not in VEGFR2–VEGFR3 heterodimers [69]. *VEGFR3^−/−^* knockout mice die during embryogenesis between E10 to E11 caused by impaired hierarchical formation of the peripheral blood vasculature and defects in cardiac remodelling [70]. The role(s) of VEGFR3 in lymphatic endothelial cell responses is well-studied; however, VEGFR3 expression is also up-regulated in vascular endothelial cells during angiogenesis [40,71]. VEGFR3 expression is also detected in non-endothelial cells, such as macrophages, neuronal progenitors and osteoblasts, whereas its functional presence in tumours is much debated [40]. Mice expressing kinase-deficient VEGFR3 maintain normal physiological blood vessel development but exhibit impaired lymphatic development [72]. VEGFR3 mutations which perturb tyrosine kinase activity are associated with variants of hereditary lymphoedema, reinforcing the pivotal role of VEGFR3 in lymphatic endothelial cell function [73].

## SIGNAL TRANSDUCTION

Most parenchymal cells express and secrete VEGF-like ligands such as VEGF-A. These ligands act in a paracrine manner on neighbouring endothelial cells to regulate VEGFR-mediated signal transduction and influence endothelial, lymphatic, epithelial and neural cell responses [1,74,75]. Notably, autocrine VEGF-A-induced signal transduction is considered essential for maintaining endothelial cell survival [76]. VEGF-stimulated signal transduction regulates a host of endothelial cell responses including proliferation, migration, permeability and cell–cell interactions.

Activation of the cytoplasmic tyrosine kinase domain by ligand-induced VEGFR2 homo- or heterodimerization causes conformational changes that expose the ATP-binding site within the tyrosine kinase domain [77]. The exchange of ADP for ATP initiates *trans*-autophosphorylation of key tyrosine residues on the receptor dimer which create docking sites for SH2-domain-containing signal adaptor molecules and trigger waves of intracellular signal transduction [78]. VEGFR tyrosine kinase activity is tightly regulated by ubiquitination, internalization, dephosphorylation and degradation by PTPs (protein tyrosine phosphatases) such as PTP1B and VE-PTP (vascular endothelial PTP) [79].

### VEGFR1-regulated signal transduction

A highly postulated model is that VEGFR1 acts as a ‘VEGF trap’ [54]. Nonetheless, VEGF-A binding to VEGFR1 Ig-like domains 2 and 3 (Figure 1) can trigger relatively low levels of *trans*-autophosphorylation on specific VEGFR1 cytoplasmic tyrosine residues Tyr^794^, Tyr^1169^, Tyr^1213^, Tyr^1242^, Tyr^1309^, Tyr^1327^ and Tyr^1333^ (Figure 2) [80–82]. Patterns of VEGFR1 tyrosine phosphorylation are ligand-dependent, e.g. VEGFR1-pY1309 epitope is caused by PlGF binding and linked to downstream activation of Akt (protein kinase B) and effects on cell physiology [83].

Computational modelling suggests VEGFR1–VEGFR2 heterodimers comprise 10%–50% of activated VEGFR complexes in response to VEGF-A; such modelling predicts low incidence of VEGFR1 homodimers when VEGFR2 levels are relatively high [84]. Functional coupling of VEGFR1 and VEGFR2 through heterodimerization and *trans*-autophosphorylation could modulate endothelial cell responses [36]. Surprisingly, transgenic mice bearing a modified *VEGFR1-TK^−/−^* locus expressing a VEGFR1 truncated protein lacking tyrosine kinase activity are viable and exhibit normal blood vessel formation during development; however, such mice exhibit defects in VEGF-A-dependent macrophage migration [85]. Other studies on heterozygous *VEGFR1-TK^+/−^* transgenic mice suggest that VEGFR1 tyrosine kinase activity is required for angiogenesis during tumour metastasis, in some inflammatory diseases, stroke, liver repair, gastric ulcer healing and various carcinomas and glioblastomas [85]. Although VEGFR1 is considered to be a ‘poor’ tyrosine kinase, therapeutics aimed at this RTK could be an attractive option for specific disease states [86].

VEGFR1 is functionally linked to endothelial cell migration and actin re-organization through RACK1 (receptor for activated C kinase 1) activation [87]. Additionally, activated VEGFR1 up-regulates uPA (urinary-type plasminogen activator) and PAI-1 (plasminogen activator inhibitor-1) levels which influence p38 MAPK regulation of actin dynamics, extracellular matrix degradation and cell migration [7,88]. VEGFR1-dependent activation of PI3K (phosphoinositide 3-kinase) is linked to endothelial cell proliferation and tubulogenesis [89]. Other targets of VEGFR1-mediated signal transduction include PLCγ1 (phospholipase Cγ1), Grb2 (growth factor receptor-bound protein 2) and PTPN11 (PTP non-receptor type 11)/SHP2 (SH2 domain-containing tyrosine phosphatase 2) [48]. VEGFR1 activation produces the cytoplasmic pY1169 and pY794 epitopes that promote PLCγ1 recruitment, leading to PIP_2_ (phosphatidylinositol-4,5-bisphosphate) cleavage and production of DAG (diacylglycerol) and IP_3_ (inositol-1,4,5-trisphosphate) [2,80]. IP_3_ binding to the membrane-bound IP_3_R (IP_3_ receptor) in the endoplasmic reticulum facilitates Ca^2+^ ion translocation into the cytosol. One consequence of such activity is engagement of the calmodulin-calcineurin pathway which causes dephosphorylation of NFAT (nuclear factor of activated T-cells) family members leading to their activation, nuclear translocation and stimulation of gene transcription at specific loci [36]. This pro-angiogenic pathway promotes an inflammatory response [90]. VEGFR1-specific ligands such as PlGF and VEGF-B bind to monocytes and stimulate intracellular signalling events including ERK1/2 (extracellular signal-regulated kinase 1/2), Akt and p38 MAPK pathways [91].

### VEGFR2-regulated signal transduction

VEGFR2-specific signal transduction influences endothelial proliferation, migration, survival and tubulogenesis. Ligand binding to the VEGFR2 extracellular domain triggers cytoplasmic tyrosine kinase activation and *trans*-autophosphorylation at residues Tyr^801^, Tyr^951^, Tyr^1054^, Tyr^1059^, Tyr^1175^, Tyr^1214^, Tyr^1223^, Tyr^1305^, Tyr^1309^ and Tyr^1319^ (Figure 2). The VEGFR2–pY951 epitope provides a binding site for SH2 domain-containing TSAd (T-cell-specific adaptor molecule) which is functionally linked to endothelial cell migration and vascular permeability [92]. Generation of the VEGFR2–pY1059 epitope enables recruitment of the proto-oncogene and soluble tyrosine kinase Src (proto-oncogene c-Src) which can further promote phosphorylation of residue Tyr^1175^. VEGFR2–pY1175 recruitment of PLCγ1 causes DAG release and activation of PKC and MAPK enzymes (e.g. ERK1/2) which influence gene expression and cell proliferation [93]. Furthermore, VEGF-A stimulates both membrane and sVEGFR1 expression through VEGFR2 and PKC-dependent pathways [94]. VEGF-A-stimulated ERK1/2 activation leads to the hyperphosphorylation of ATF-2 (activating transcription factor 2), causing elevated expression of VCAM-1 (vascular endothelial cell adhesion molecule 1) and promoting endothelial-leucocyte interactions [95]; this now provides a MAPK-regulated gene expression mechanism that links angiogenesis and inflammation.

VEGFR2–pY1175 recruitment of PLCγ1 and adaptor protein Shb (SH2-domain-containing adaptor protein B) facilitates interaction with FAK (focal adhesion kinase) and contributes to endothelial cell migration and attachment [96]. Shb activation of PI3K results in sequential activation of Akt and eNOS (endothelial nitric oxide (NO) synthase) which promote cell survival and NO-induced vascular permeability respectively [2,51]. The VEGFR2–pY1214 epitope recruits the adaptor protein Nck (Nck adaptor protein 1) and a cytoplasmic tyrosine kinase, Fyn (Fyn proto-oncogene, Src family tyrosine kinase) Nck–Fyn complex formation regulates phosphorylation of PAK2 (p21-activated protein kinase 2), which in turn activates Cdc42 (cell division cycle 42) and p38 MAPK [97]; impacting on cell migration through increased actin remodelling. VEGF-regulated PI3K activation mediates cell survival through sequential PDK1 (PI-dependent protein kinase 1) and Akt activation. Akt is a multi-functional regulator that can target BAD (Bcl-2-associated agonist of cell death) and caspase 9, thus blocking apoptosis [98].

Other post-translational modifications, such as methylation, are involved in VEGFR2 activation. VEGFR2 methylation takes place at multiple lysine and arginine residues, such as residue Leu^1041^ which is proximal to the kinase domain activation loop. Although methylation is ligand-independent, it enhances tyrosine phosphorylation and kinase activity in response to ligand [99]. In addition, VEGFR2 is acetylated at a dense cluster of four lysine residues in the kinase insert domain and at a single lysine within the kinase activation loop [100]. The acetyltransferase p300 and two deacetylases, histone deacetylase 5 (HDAC5) and HDAC6, regulate VEGFR2 acetylation in a process essential for controlling sustained ligand-dependent RTK *trans*-autophosphorylation and downstream signal transduction [100].

### VEGFR3-regulated signal transduction

VEGF-C or VEGF-D binding to VEGFR3 stimulates tyrosine kinase activation and phosphorylation of VEGFR3 cytoplasmic domain residues Tyr^830^, Tyr^833^, Tyr^853^, Tyr^1063^, Tyr^1068^, Tyr^1230^, Tyr^1231^, Tyr^1265^, Tyr^1333^, Tyr^1337^ and Tyr^1363^ (Figure 2) [69]. The VEGFR3–pY1063 epitope facilitates interaction with CRK I/II (adaptor protein C10 regulator of kinase) which in turn activates the JNK (c-Jun N-terminal kinase) pathway and promotes cell survival [101]. PI3K activation is crucial for lymphatic development by mediating lymphoendothelial migration [102]. Ligand-induced phosphorylation of VEGFR3 residues Tyr^1230^ and Tyr^1232^ regulates cell migration, apoptosis and lymphoendothelial cell DNA synthesis [101]. The VEGFR3–pY1230 and–pY1232 epitopes recruit the Shc and Grb2 adaptor proteins which promote signal transduction through ERK1/2 and Akt/PI3K pathways which are important for lymphatic endothelial cell migration [101,103]. Formation of VEGFR2–VEGFR3 heterodimers is required for VEGF-C-induced Akt activation. In contrast, ERK1/2 is activated by VEGFR3 homodimers [103]. VEGFR2–VEGFR3 heterodimers are common in endothelial cells engaged in active angiogenesis, particularly at tip cell filopodia. Although VEGF-A does not bind VEGFR3, it is possible that VEGFR3 contributes to VEGF-A mediated blood vessel sprouting through heterodimer formation [77].

## VEGFR TRAFFICKING AND TURNOVER

VEGFR1 was initially postulated solely as a plasma membrane resident RTK but work has revealed that ∼80% is located within a stable pool in the Golgi apparatus along the secretory pathway [104]. VEGF-A stimulation of endothelial cells causes transient redistribution of VEGFR1 to the plasma membrane via a *trans*-Golgi network-to-plasma membrane route that is regulated by cytosolic calcium flux [104] (Figure 3). Calcium ion-regulated trafficking involves activation of VEGFR2 at the plasma membrane; this feedback model explains how VEGF-A-stimulated cellular responses are linked to VEGFR1 and VEGFR2 trafficking and turnover [105]. VEGFR1 levels are relatively insensitive to VEGF-A stimulation and endosome-lysosome trafficking, unlike VEGFR2 [88,104]. Activated VEGFR1 is internalized through clathrin-mediated endocytosis by ternary complex formation with CBL (Cas-Br-M murine ecotropic retroviral transforming sequence homologue) [106] and adaptor protein CD2AP (CD2-associated protein), followed by association with clathrin [107].

**Figure 3:**
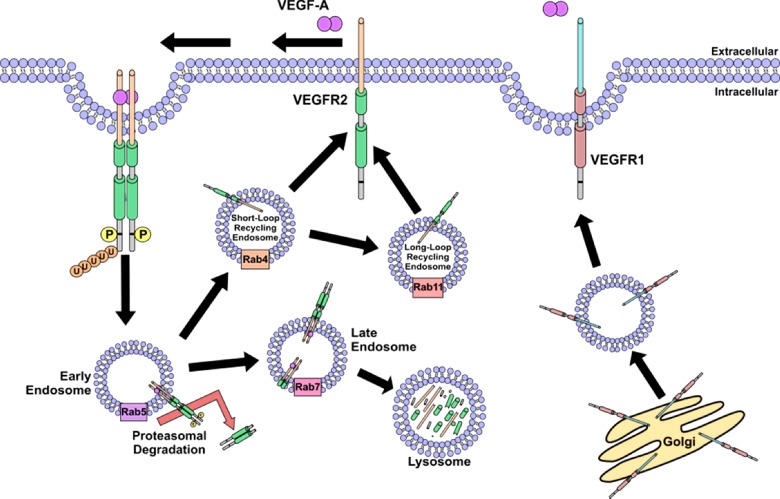
VEGFR activation, trafficking and proteolysis Schematic depicting ligand-activated VEGFR trafficking and proteolysis. Upon VEGF-A binding, VEGFR2 undergoes dimerization, trans-autophosphorylation and ubiquitination. Following internalization into early endosomes, both activated and quiescent VEGFR2 can undergo recycling back to the plasma membrane via short- or long-loop recycling pathways. Alternatively, ubiquitinated VEGFR2 undergoes 26S proteasome-regulated cleavage of its C-terminus in early endosomes followed by trafficking to late endosomes and lysosomes for terminal degradation. VEGF-A-stimulated VEGFR2 signal transduction promotes increased calcium-dependent plasma membrane translocation of VEGFR1 thus creating a negative feedback loop to attenuate VEGFR2 activity.

VEGFR2 is localized to the Golgi, plasma membrane, early endosomes and perinuclear caveolae in non-stimulated endothelial cells [105,108–110]. Resting VEGFR2 is distributed between the plasma membrane (∼40%) and an internal early endosomal pool (∼60%), with constitutive recycling between the two compartments [108,111,112]. Recent work has revealed requirement for syntaxin 6 [109] and the kinesin motor protein KIF13B (kinesin family member 13B) [113] in biosynthetic VEGFR2 trafficking through the Golgi apparatus *en route* to the plasma membrane. Resting VEGFR2 undergoes a relatively fast rate of ligand-independent, constitutive internalization [111,114] which does not require tyrosine kinase activity [108]. Phosphorylation of residues Tyr^1054^ and Tyr^1059^ is required for clathrin-dependent internalization of activated VEGFR2 [115]. Chaperone proteins such as HSP70 (heat shock protein 70) have been linked to VEGFR2 ubiquitination and trafficking [116]. HSP70 is associated with VEGFR2 degradation following clathrin-mediated endocytosis. In contrast, HSP90 stabilizes VEGFR2 levels [116]. Thus, the HSP70–HSP90 axis is essential for regulating VEGFR2 homoeostasis. Another chaperone protein involved in VEGFR2 stabilization is PDCL3 (phosducin-like 3). Receptor ubiquitination and degradation is inhibited by PDCL3 binding to the juxtamembrane domain of VEGFR2 thus increasing VEGF-A-stimulated tyrosine phosphorylation [117].

VEGF-A-activated VEGFR2 undergoes endocytosis and targeting for either recycling or degradation [118] (Figure 3). VEGF-A stimulation promotes ∼40%–60% degradation of plasma membrane and endosomal VEGFR2 pools [111]. Ligand binding causes a redistribution of VEGFR2 from early to late endosomes depending upon VEGF-A concentration and duration of stimulation; nonetheless, a significant early endosomal pool of VEGFR2 is maintained [111]. It has been proposed that the small Rab (Ras-associated protein) GTPase family members, Rab5a and Rab7a, have regulatory roles in VEGFR2 trafficking and signal transduction in early and late endosomes respectively [119,120] (Figure 3). Following VEGF-A stimulation, phosphorylated and ubiquitinated VEGFR2 is transported to early endosomes after recognition by the ubiquitin-linked receptor complex, ESCRT-0 (endosomal sorting complex required for transport) [121]. Early endosomal localization of VEGFR2 is essential for maximal activation of Akt and ERK1/2 signal transduction pathways [122,123]. In contrast, p38 MAPK signal transduction is linked to cell surface VEGFR2 [31,114,124].

Recycling of activated VEGFR2 occurs through Rab4a- or Rab11a-positive endosomes and follows a short loop (Rab4a) or long loop (Rab11a) pathway [112,125] (Figure 3). Long loop recycling occurs in co-ordination with NRP1 trafficking following transition from Rab4a-postive vesicles. Rab4a–Rab11a transition is co-ordinated by interaction between the C-terminal PDZ-binding motif of synectin, myosin VI and the NRP-1 C-terminal motif, SEA (serine-glutamic acid-alanine) [125–128]. Receptor recycling via Rab11a-positive endosomes is VEGF-A isoform-dependent. For example, VEGF-A_165b_ isoform is unable to bind NRP1 and fails to promote Rab11a-dependent recycling [125].

Internalized VEGFR2 continues to signal from multiple cellular compartments until it is committed for recycling or degradation [129]. Internalized VEGFR2 is directed for lysosomal degradation as a result of ubiquitination by E3 ligases c-Cbl (Cbl proto-oncogene E3 ubiquitin protein ligase) or β-TrCP1 (β-Transducin repeat containing E3 ubiquitin protein ligase) [106,130,131]. VEGF-A-stimulated ubiquitination or activation of non-classical PKC isozymes promotes increased trafficking and proteolysis of VEGFR2 in the endosome–lysosome system [88,119,130,132]. VEGFR2 proteolysis is tightly regulated; at least two distinct proteolytic steps have been postulated for processing within the endosome-lysosome system. A 26S proteasome-regulated step is associated with early endosomes and cleavage of the C-terminal domain prior to lysosomal processing of the extracellular/luminal domain [88,130] (Figure 3). Proteasome-mediated VEGFR2 proteolysis regulates VEGFR2 signal transduction through the Akt, eNOS and MAPK pathways [130].

## DRUGS AND DISEASE

Pathological angiogenesis is associated with multiple diseases. Neurodegenerative disorders, pulmonary hypertension and ischaemic events are associated with inadequate angiogenesis. In contrast, excessive angiogenesis occurs in disorders characterized by abnormal vascular growth and remodelling such as cancer, infectious and inflammatory diseases and AMD [133]. Anti-angiogenic therapy has been targeted towards members of the VEGF family and associated VEGFRs due to their essential role in angiogenesis [134] (Figure 4). Bevacizumab (Avastin) is a humanized monoclonal antibody to VEGF-A approved to treat RCC (renal cell carcinoma), metastatic colorectal cancer, metastatic breast cancer, advanced non-squamous, non-small cell lung cancer and recurrent glioblastoma multiforme [135]. Aflibercept (Zaltrap, VEGF Trap-Eye) is a recombinant fusion protein consisting of the extracellular VEGF-A-binding domains of VEGFR1 and VEGFR2 fused to an Fc domain. This new anti-angiogenic molecule acts as a decoy receptor to block VEGF-A, VEGF-B and PlGF activity and has been approved for the treatment of metastatic RCC [136] (Figure 4).

**Figure 4:**
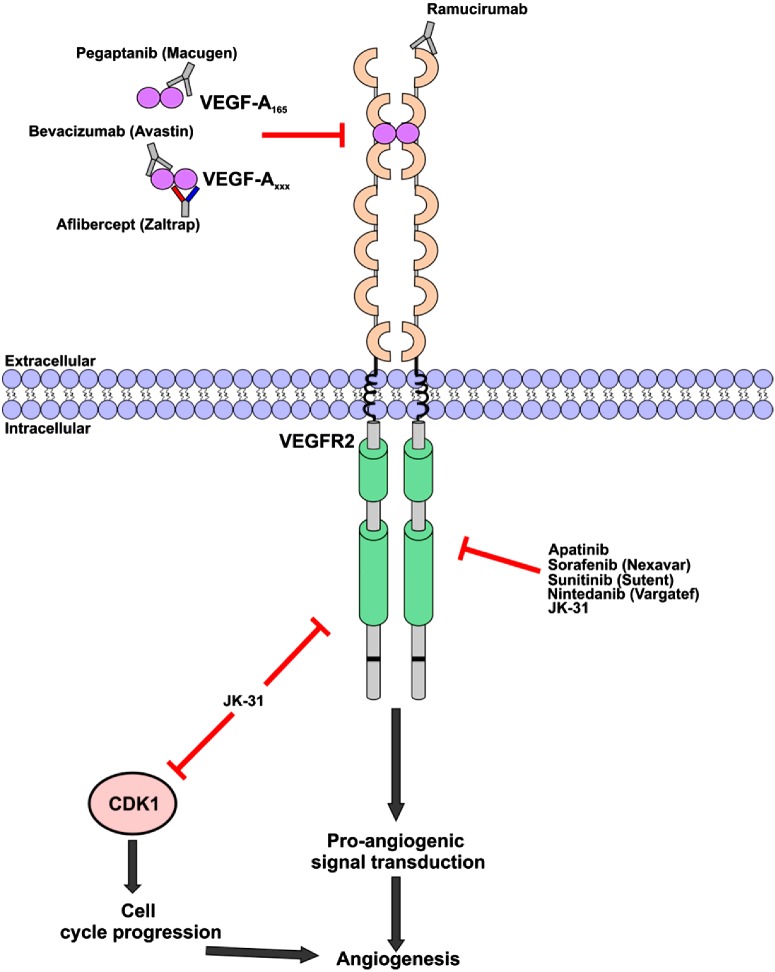
Therapeutic inhibitors of VEGFR2 signal transduction Schematic depicting target sites of anti-angiogenic agents that inhibit VEGFR2-mediated signal transduction. VEGF-A_xxx_; non-specific VEGF-A isoform.

TKIs (tyrosine kinase inhibitors) constitute another class of anti-angiogenic drugs approved for cancer therapy. These inhibitors disrupt VEGFR1 and/or VEGFR2 signal transduction and often interfere with the activity of other receptor tyrosine kinases such as FGF (fibroblast growth factor) and PDGF receptors [137,138]. The most successful VEGF-related therapies which provide greatest improvement in progression-free survival in cancer patients include Sorafenib and Sunitinib [139] (Figure 4). Sorafenib (Nexavar) is a TKI approved for the treatment of metastatic RCC and hepatocellular carcinoma [140]. The anti-cancer drug Sunitinib (Sutent) is a member of the indolinone family of compounds and is approved to treat RCC and gastrointestinal stromal tumour [140]. Although providing short-term benefits, the activity of these drugs is limited by the introduction of compensatory pathways or resistance mechanisms [138,141]. For example, increased hypoxia as a consequence of VEGF-A inhibition up-regulates pro-angiogenic factors, such as FGFs and PlGF, and promotes recruitment of pro-angiogenic bone marrow-derived cells to induce tumour revascularization [142]. Maintaining the correct balance of inhibition of a select group of receptor tyrosine kinases including VEGFRs and FGF receptors (FGFRs) thus appears clinically important [138,141]. One strategy to combat drug resistance to VEGF inhibitors is the development of multi-targeted TKIs. For example, Nintedanib (Vargatef) is a small molecule multi-target TKI of FGFR, PDGFR and VEGFR used in the treatment of non-small-cell lung cancer [143]. JK-31 is a multi-kinase inhibitor that targets VEGFR2 and CDK1 (cyclin-dependent kinase 1) to simultaneously inhibit pro-angiogenic signal transduction and cell cycle progression in endothelial cells [144]. Another multi-targeted TKI JK-P3 inhibits the intrinsic catalytic activity of VEGFR2 (Figure 4), FGFR1) and FGFR3 [145]. The ability of multi-targeted TKIs to simultaneously inhibit multiple signal transduction pathways enables them to overcome redundant angiogenic factors considered to be a key mechanism underlying resistance to anti-VEGF therapy [146].

The importance of anti-angiogenic drugs in cancer therapy has been highlighted by their use to treat nine distinct solid tumours. Emerging anti-angiogenic agents that selectively inhibit VEGFR2 and VEGFR2-related signalling include Ramucirumab, a fully humanized monoclonal antibody targeting the extracellular domain of VEGFR2 and Apatinib, a small molecule inhibitor of the intracellular domain [140]. Advancement in anti-angiogenic therapies is necessary since they currently prolong survival of responsive patients by months rather than providing long-term progression-free survival and are often only effective in combination with chemotherapy [142].

High levels of VEGF-A found in ocular fluid are associated with AMD, diabetes and ischaemic central retinal vein occlusion [142]. Interestingly, the levels of different VEGF-A isoforms in extracellular fluids could be associated with disease states [17]. In this context, increased expression of the VEGF-A_165b_ isoform is associated with altered risk of prostate cancer and peripheral arterial disease [147,148]. Current treatments that directly target circulating VEGF-A in diseases such as wet AMD include Pegaptanib (Macugen), a pegylated 28-base ribonucleic aptamer that selectively binds the heparin-binding domain of VEGF-A_165_ and inhibits bioactivity (Figure 4). Ranibizumab (Lucentis) is a recombinant, humanized anti-VEGF-A antibody fragment derived from Bevacizumab, also approved to treat AMD. Ranibizumab is smaller than Bevacizumab and is thought to deliver more effective retinal penetration [142]. The use of anti-VEGF drugs as monotherapy for the treatment of AMD has proved successful with increased visual acuity experienced by 30% of patients [142].

VEGF-C-induced VEGFR3 activity has been linked to cancer metastasis and disease progression in lung cancer patients [149]. Peptides developed to inhibit the kinase activity of VEGFR3 suppress VEGF-C-mediated cancer cell invasiveness and VEGF-C-induced drug resistance by inhibiting VEGFR3-linked signal transduction [149]. In addition to anti-angiogenic agents, it is desirable that pro-angiogenic drugs will be developed as high impact therapies for cardiovascular diseases.

Current VEGFR therapies target cancer or AMD however future research directions for VEGFR-targeted therapeutics are emerging for other diseases. VEGF plays a crucial protective role in the nervous system. Reduced levels of VEGF and other growth factors are associated with neurodegenerative diseases. VEGF has been identified as a causative factor in several motor neuron degenerative diseases [150] and epilepsy [151]. VEGF is of particular interest due to its role in cross-talk between the nervous and vascular systems. These multi-tasking effects of VEGF make it a promising therapeutic target.

## CONCLUDING REMARKS

The VEGF–VEGFR axis is essential for the maintenance of endothelial and vascular homoeostasis and function. A switch to pro- or anti-angiogenic outcomes is associated with diseases ranging from cancer to chronic inflammation and diabetes mellitus. The VEGF–VEGFR system generates a diverse array of responses in different cell types and tissues through VEGF isoform-mediated RTK dimerization and co-receptor recruitment. Emerging research is uncovering essential roles for VEGF–VEGFR signal transduction in non-vascular tissues including the epithelium, brain and immune system. Complexity in the response of cells and tissues to VEGF isoforms highlights the therapeutic potential of manipulating this receptor–ligand axis. A challenge is to target specific VEGF or VEGFR isoforms using antibody, protein or gene-based strategies to shed light on mechanistic principles. Such work could also lay the foundations for more selective drug targeting in disease therapy. VEGF–VEGFR biology is an exciting and expanding area of biology and medicine that is generating new insights into molecular and cellular mechanisms that are being translated into the next generation of therapeutics for major diseases.

## Abbreviations

Akt: protein kinase B
AMD: age-related macular degeneration
CBL: Cas-Br-M murine ecotropic retroviral transforming sequence homologue
DAG: diacylglycerol
eNOS: endothelial nitric oxide synthase
ERK1/2: extracellular signal-regulated kinase 1/2
FGF: fibroblast growth factor
FGFR: fibroblast growth factor receptor
Flt: fms-related tyrosine kinase
Fyn: Fyn proto-oncogene, Src family tyrosine kinase
Grb2: growth factor receptor-bound protein 2
HDAC: histone deacetylase
HSP: heat shock protein
HS: heparan sulfate
HSPG: heparan sulfate glycoprotein
IP_3_: inositol-1,4,5-trisphosphate
MAPK: mitogen-activated protein kinase
Nck: Nck adaptor protein 1
NO: nitric oxide
NRP: neuropilin
PDCL3: phosducin-like 3
PDGF: platelet-derived growth factor
PI3K: phosphoinositide 3-kinase
PKC: protein kinase C
PLCγ1: phospholipase Cγ1
PlGF: placental growth factor
PTP: protein tyrosine phosphatase
Rab: Ras-associated protein
RCC: renal cell carcinoma
RTK: receptor tyrosine kinase
Shb: SH2-domain-containing adaptor protein B
sVEGFR: soluble VEGFR
TACE: tumour necrosis factor α converting enzyme
TKI: tyrosine kinase inhibitor
VEGF: vascular endothelial growth factor
VEGFR: vascular endothelial growth factor receptor tyrosine kinase
